# The bacteriocin Angicin interferes with bacterial membrane integrity through interaction with the mannose phosphotransferase system

**DOI:** 10.3389/fmicb.2022.991145

**Published:** 2022-09-06

**Authors:** Verena Vogel, Lia-Raluca Olari, Marie Jachmann, Sebastian J. Reich, Michelle Häring, Ann-Kathrin Kissmann, Frank Rosenau, Christian U. Riedel, Jan Münch, Barbara Spellerberg

**Affiliations:** ^1^Institute of Medical Microbiology and Hygiene, Ulm University Medical Center, Ulm, Germany; ^2^Institute of Molecular Virology, Ulm University Medical Center, Ulm, Germany; ^3^Institute of Microbiology and Biotechnology, University of Ulm, Ulm, Germany; ^4^Institute of Pharmaceutical Biotechnology, University of Ulm, Ulm, Germany

**Keywords:** Angicin, *Streptococcus anginosus*, mannose phosphotransferase system (Man-PTS), bacteriocin, receptor, mode of action (MOA)

## Abstract

In a natural environment, bacteria are members of multispecies communities. To compete with rival species, bacteria produce antimicrobial peptides (AMPs), called bacteriocins. Bacteriocins are small, cationic, ribosomally synthesized peptides, which normally inhibit closely related species of the producing organism. Bacteriocin production is best studied in lactic bacteria (LAB). *Streptococcus anginosus*, belonging to LAB, produces the potent bacteriocin Angicin, which shows inhibitory activity against other streptococci, *Listeria monocytogenes* and vancomycin resistant *Enterococcus faecium* (VRE). Furthermore, Angicin shows a high resistance toward pH changes and heat, rendering it an interesting candidate for food preservation or clinical applications. The inhibitory activity of Angicin depends on the presence of a mannose phosphotransferase system (Man-PTS) in target cells, since *L. monocytogenes* harboring a deletion in an extracellular loop of this system is no longer sensitive to Angicin. Furthermore, we demonstrated by liposome leakage and pHluorin assays that Angicin destroys membrane integrity but shows only low cytotoxicity against human cell lines. In conclusion, we show that Angicin has a detrimental effect on the membrane of target organisms by using the Man-PTS as a receptor.

## Introduction

It is a common trait of lactic acid bacteria (LAB) to produce bacteriocins, and numerous streptococcal bacteriocins have been described (Barbour et al., 2013; Soto et al., 2017; Hertzog et al., 2018; Vogel and Spellerberg, 2021). We recently identified the novel bacteriocin Angicin of *Streptococcus anginosus* (Vogel et al., 2021).

As a conserved defense mechanism against infections, most living organisms produce antimicrobial peptides (AMPs) (Chikindas et al., 2018). Bacteriocins, the AMPs produced by bacteria, often inhibit only the growth of closely related species however, some bacteriocins also have a broader spectrum of activity (McAuliffe et al., 1998; Rea et al., 2010, 2011). These ribosomally produced antimicrobials give the producing organism a colonization advantage over competitors (Heilbronner et al., 2021). AMP classification distinguishes between bacteriocins that undergo major post-translational modifications (class I) and nearly unmodified peptides (class II) (Alvarez-Sieiro et al., 2016). Class II bacteriocins are further classified in pediocin-like (subclass IIa), two-peptides (subclass IIb), leaderless (subclass IIc) and non-pediocin-like, single bacteriocins (subclass IId) (Alvarez-Sieiro et al., 2016). Typical characteristics of bacteriocins are a size below 10 kDa, high thermal and pH stability. For bacteriocins not only an antibacterial activity was described, but some bacteriocins have additional antiviral and antifungal functions (Al Kassaa et al., 2014; Dehghanifar et al., 2019; de Souza de Azevedo et al., 2020; Kim et al., 2020). In addition, anticancer and immunomodulatory effects of bacteriocins have been reported (Joo et al., 2012; Małaczewska et al., 2019). Additionally, bacteriocins are colorless, odorless and tasteless (Perez et al., 2014). Such properties combined with antimicrobial activity render bacteriocins interesting candidates for a variety of applications (Negash and Tsehai, 2020). For example, the most prominent bacteriocin, Nisin (commercially available as Nisaplin™) produced by *Lactococcus lactis*, is approved as a compound for food preservation by the European Food Safety Authority (E number: E234) and the U.S. Food and Drug administration (Title 21 of the Code of Federal Regulations § 184.1538). Furthermore, several other bacteriocins or bacteriocin producing strains are applied in food preservation, including the bacteriocin Pediocin PA-1 (Alta 2341™), the sakacin producer *Lactobacillus sakei* (Bactoferm™ B2) or the leucocin producer *Leuconostoc carnosum* (Bactoferm™ B-SF-43) (Rodríguez et al., 2002; Silva et al., 2018; Abdulhussain Kareem and Razavi, 2020; Daba and Elkhateeb, 2020). With the ongoing increase of (multi)drug resistant bacteria and the decrease of available and applicable antibiotics, bacteriocins are also investigated as antimicrobial therapeutics (Duraisamy et al., 2020; Hu et al., 2020; Walsh et al., 2021). They have already been successfully tested for a topical application against skin infections caused by methicillin resistant *Staphylococcus aureus* (MRSA), against oral infectious diseases and for the treatment of *Clostridium difficile* infections (Rea et al., 2011; Tong et al., 2014; Ovchinnikov et al., 2020).

*Streptococcus anginosus* belongs to the family of LAB, and together with *Streptococcus constellatus* and *Streptococcus intermedius* it forms the *Streptococcus Anginosus* Group (SAG). It is found as a colonizer of mucosal membranes like the oral cavity, gastrointestinal and urogenital tract (Poole and Wilson, 1979; Whiley et al., 1992). The pathogenic potential of *S. anginosus* has been underestimated in the past, but this species can cause severe infections at all body sites. Isolation from abscesses, urine, blood cultures and cystic fibrosis patients has been reported and the incidence rate of invasive SAG infections is higher than the incidence rate for *Streptococcus pyogenes* and *Streptococcus agalactiae* combined (Laupland et al., 2006; Reissmann et al., 2010; Sibley et al., 2010; Kobo et al., 2017; Jiang et al., 2020). Therefore, exploring factors involved in the development of *S. anginosus* infections is important.

Angicin is a class IId bacteriocin produced by *S. anginosus* (Vogel et al., 2021). Mature Angicin has a molecular mass of 6053.1 Da and consists of 54 amino acids. A respective leader peptide with a double glycine motif is presumably cleaved of during processing and export. Angicin is active against closely related streptococci, listeria and vancomycin resistant *Enterococcus faecium* (VRE). While Angicin has been shown to disrupt membrane integrity of *Listeria monocytogenes* a receptor has not been identified (Vogel et al., 2021).

Pore formation is the most common antimicrobial mechanism of bacteriocins. These peptides are cationically charged, which allows an efficient interaction with the negatively charged bacterial membrane (Vasilchenko and Valyshev, 2019). Pore formation in the bacterial membrane leads to the disruption of the proton motive force and leakage of intracellular substrates, eventually resulting in cell death (van Belkum et al., 1991; Christensen and Hutkins, 1992; Minahk et al., 2000). Many bacteriocins have been shown to act via a receptor-dependent mechanism (Breukink et al., 1999; Cotter, 2014; Jeckelmann and Erni, 2020; Tymoszewska et al., 2020). For serval class II bacteriocins a wide range of activity in the nanomolar range has been reported and the mannose phosphotransferase system (Man-PTS) has been identified as receptor (Ramnath et al., 2004; Diep et al., 2007; Iwatani et al., 2007; Tosukhowong et al., 2012; Ríos Colombo et al., 2018; Tymoszewska et al., 2020; Oftedal et al., 2021). The Man-PTS is responsible for mannose and glucose uptake with a simultaneous phosphorylation of the sugar (Simoni et al., 1976; Jeckelmann and Erni, 2020).

For future applications of Angicin either as a food preservative or in a clinical setting further information on receptor, mechanism of action and cytotoxicity is crucial. To further explore its range of activity we wanted to know, whether Angicin, like other bacteriocins, is able to not only inhibit bacteria but also viruses and fungi. Such activity would broaden the range of applications for Angicin. As a novel finding of our study, the Man-PTS was identified as the receptor for Angicin. By liposome leakage and pHluorin assays, membrane disruption was established as the mode of action used by Angicin. Furthermore, we show that Angicin is not cytotoxic. Angicin shows a specific activity against bacteria, while viruses and fungi are not affected.

## Materials and methods

### Bacterial strains and growth conditions

All strains used in this study (Table 1) were cultivated on sheep blood agar plates (Oxoid, Basingstoke, United Kingdom) and incubated at 37°C and 5% CO_2_. For all non- *Escherichia coli* bacteria, liquid cultivation was performed in Todd-Hewitt Broth (Oxoid) supplemented with 0.5% yeast extract (THY, Gibco, Waltham, MA) under the same conditions. For *E. coli*, liquid cultivation was performed in lysogeny broth (LB-Miller) at 37°C while shaking (180 rpm). *Listeria monocytogenes* harboring pNZ44 or its derivates was cultivated in brain-heart-infusion medium (Oxoid) supplemented with 10 μg/ml chloramphenicol (Sigma-Aldrich Chemie GmbH, Steinheim am Albuch, Germany) at 37°C while shaking (180 rpm).

**TABLE 1:** All bacterial and fungal strains and plasmids used in this study.

Strain or plasmid	Definition	Source
**Bacteria**		
*Escherichia coli* DH5α	endA1 hsdR17 supE44 DlacU169(f80lacZDM15) recA1 gyrA96 thi-1 relA1	Boehringer
*Streptococcus anginosus* BSU 1211	*S. anginosus*, clinical isolate	Bauer et al., 2020
*Listeria monocytogenes* EGDe	Ln II Serotype I/2a	Bécavin et al., 2014
*Listeria monocytogenes* EGY2	EGDe derivative carrying a deletion of 84 bp in the mptD gene	Dalet et al., 2001
*Listeria monocytogenes* pNZ-pHin2*^LM^*	*L. monocytogenes* EGDe carrying pNZ-pHin2*^LM^*	Reich et al., 2022
*Listeria ivanovii* CIP 78.42T	–	Zetzmann et al., 2015
*Listeria grayi* CIP 68.18T	–	Zetzmann et al., 2015
*Enterococcus faecium* BSU 1516	VRE, DSM 17050	DSM
*Pseudomonas aeruginosa BSU 856*	ATCC 27853	ATCC
*Staphylococcus aureus* BSU 1348	MRSA, ATCC 43300	ATCC
**Plasmids**		
pNZ44	*E. coli-L. lactis* high-copy-number shuttle vector, Cm^r^, constitutive P44 promoter from *L. lactis*	McGrath et al., 2001
pNZ-pHin2*^LM^*	pnZ44 derivate, high level constitutive expression of pHin2*^LM^*	Reich et al., 2022
**Fungi**		
*Candida albicans*	ATCC 90028	IPK laboratory of medical mycology
*Candida auris*	DSM 21092	IPK laboratory of medical mycology
*Candida parapsilosis*	ATCC 22019	IPK laboratory of medical mycology

### Human cell lines and culture conditions

Vero E6 (*Cercopithecus aethiops* derived epithelial kidney) cells were purchased from ATCC^®^ and grown in Dulbecco’s modified Eagle’s medium (DMEM, Gibco) which was supplemented with 2.5% heat-inactivated fetal calf serum (FCS), 100 units/ml penicillin, 100 μg/ml streptomycin, 2 mM L-glutamine, 1 mM sodium pyruvate, and 1x non-essential amino acids. ELVIS cells (Enzyme-Linked Virus-Inducible System—ELVIS™), also from ATCC^®^ are genetically engineered baby hamster kidney cells that encode a lacZ gene, which is expressed upon infection via the viral transactivator ICP10 (Proffitt and Schindler, 1995). TZM-bl cells are HeLa derived cell line expressing CD4, CCR5 and CXCR4, encoding luciferase and β-galactosidase genes under the control of the HIV-LTR promoter (Wei et al., 2002). ELVIS and TZM-bl cells were grown in DMEM supplemented with 2 mM L-glutamine, 100 units/ml penicillin, and 100 μg/ml streptomycin and 10% heat-inactivated FCS. Monocytic THP-1 cells were cultivated in Roswell Park Memorial Institute 1640 medium (RPMI 1640, Gibco™ RPMI 1640 Medium, GlutaMAX™, Life Technologies Limited) supplemented with 0.01 M HEPES Buffer (PAN-Biotech, Aidenbach, Germany), 10% v/v fetal bovine serum (FBS superior stabil, Bio&Sell GmbH, Feucht, Germany) and 0.2% v/v 2-mercaptoethanol (SERVA Electrophoresis GmbH, Heidelberg, Germany). Cells were incubated at 37°C and 5% CO_2_.

### Culture conditions *Candida*

*Candida albicans, Candida parapsilosis*, and *Candida auris* were cultured on Sabouraud dextrose agar (40 g/L glucose, 10 g/L peptone, 20 g/L agar, pH 5.6). For suspension cultures, individual colonies were inoculated in shaking flasks with 10 ml of RPMI-1640 supplemented with 300 mg/l L-glutamine (Thermo Fisher Scientific, Waltham, United States) and grown at 37°C with orbital shaking at 150 rpm for 16 h.

### Survival assay

Overnight cultures of *L. monocytogenes, Listeria ivanovii*, or *Enterococcus faecium* were inoculated in 10 ml THY at an O.D. _600 nm_ of 0.02. When they reached an O.D. _600 nm_ of 0.1, 1 ml was transferred in an Eppendorf tube. Subsequently, the bacterial cells were centrifuged at 8,800 × g for 2 min and the supernatant was discarded. The pellet was reconstituted in 10 mM phosphate buffer (Sigma-Aldrich) containing either 1.56 μg/ml Angicin (synthesized by PSL Heidelberg, purity > 98%) or as a control the same amount of water. Bacteria were incubated at 37°C and an aliquot was plated after 30, 60, 90, and 180 min. To determine the number of bacteria in the starting solution, water treated cells were plated after 0 min. Colony forming units per ml were determined after overnight incubation at 37°C and 5% CO_2_. At least five independent experiments were performed with technical duplicates.

### pHluorin-assay

Overnight cultures of pHluorin2-expressing *L. monocytogenes* (Reich et al., 2022) were adjusted to an O.D. _600 nm_ of 3 in *Listeria* minimal buffer, pH 6.2 (LMB) (Crauwels et al., 2018) and 50 μl of bacteria were mixed with 50 μl of LMB containing different Angicin concentrations, ranging from 10 to 0.04 μg/ml. After 30 min incubation in the dark the emission at 520 nm was determined after excitation at 400 and 480 nm using an infinite M200 microplate reader (Tecan group Ltd., Männedorf, Switzerland). The ratio of 400 nm to 480 nm was calculated. As negative and positive controls, LMB and Nisin (10 μg/ml, Sigma-Aldrich) was used, respectively.

### Liposome assay

#### Folch extraction

Lipids were extracted from live bacteria (*E. coli, Pseudomonas aeruginosa*, *L. monocytogenes*, VRE, and MRSA) or Vero E6 eukaryotic cells using the Folch method (Folch et al., 1957). Briefly, bacterial cells grown in overnight cultures (16–18 h) or Vero E6 cells from a confluent T-175 cm3 cell-culture flask were harvested by centrifugation and resuspended in 1 ml of 2:1 (v/v) chloroform/methanol mixture and vortexed 5 × 1 min. Then, 200 μl dH_2_O were added and the samples were centrifuged 7 min at 1,000 × g to induce phase separation. The lower phase containing the lipids was carefully extracted and moved in a glass vial. Liquid was removed by drying under nitrogen steam. Lipid amount was quantified by measuring the glass vial before and after the addition of lipids.

#### Liposome dye leakage

Liposome leakage assay was performed as previously described (Weil et al., 2020). Liposomes for dye-leakage assay were prepared by thin-film hydration and extrusion. Lipids previously extracted or commercially purchased (*E. coli* polar extract, Avanti polar lipids, Inc., Alabaster, AL, United States) were hydrated by adding 1 ml 50 mM 5(6)-carboxyfluorescein prepared in 50% phosphate buffered saline (PBS, resulting in a solution isoosmolar to PBS) and adjusted to pH 7.4 with NaOH, yielding a total lipid concentration of 5 mM. The glass vials were shaken at 70°C, 180 rpm, for 3 h. Small unilamellar vesicles were then prepared by 25x extrusion through 0.2 μM polycarbonate membranes (Nuclepore Track-Etched Membrane, Whatman plc, Maidstone, United Kingdom) in a Mini Extruder (Avanti Polar Lipids) on a heating platform at 70°C. Free dye was removed by 2x size-exclusion filtration using PD midiTrap Sephadex G-25 columns (GE Healthcare, Chicago, IL) and liposomes then quantified by nanoparticle tracking analysis using a ZetaView (ParticleMetrix, Inning am Ammersee, Germany). For assays in 96-well format, liposome preparations were diluted in PBS and 1–2 × 10^9^/well added to plates in 80 μl volume. Fluorescence intensity was read in a Synergy plate reader (Biotek, Winooski, VT). Baseline was established by measuring fluorescence for 5 min, 20 μl of compounds then added and plates incubated for 1 h at 37°C with measurements every 1 min. Maximum intensity (100% dye release) was then measured by adding Triton X-100 to 2% final concentration and again measuring for 5 min. As a positive control, LL-37 (AnaSpec Inc., Fremont, CA) was used. Background signal (signal before the addition of compound) was subtracted from the data and subsequently it was normalized to the maximum intensity (signal after complete liposome leakage).

### Radial diffusion assay

To asses bacteriocin activity a two-layer radial diffusion assay (RDA) was used, as described previously (Vogel et al., 2021). In short, overnight cultures of putative target organisms were washed with 10 mM phosphate buffer (Sigama-Aldrich) and the O.D. _600 nm_ was determined. Putative target strains were inoculated into warm liquid 1% agarose (Sigma-Aldrich) at a density of 2 × 10^7^ bacterial cells per plate. After solidification wells were put into the agarose plate with wide bore pipette tips (Axygen—a corning brand, Corning Inc., Corning, NY) and filled with the test substance. Following a 3 h incubation period at 37°C, an overlay with trypticase soy agar (Oxoid) was performed. Inhibition zones were measured in cm after overnight incubation at 37°C and 5% CO_2_.

For the investigation of antimicrobial activity of *S. anginosus* BSU 1211 against *L. monocytogenes* or *L. monocytogenes*Δ*mptD* a one-layer RDA was conducted (Vogel et al., 2021). Therefore, the target strains, were inoculated into liquid trypticase soy agar with a density of 2 × 10^7^ bacterial cells per plate. Wells were put into the solidified agar and filled with 10 μl of *S. anginosus* solved in 10 mM phosphate buffer and an O.D. _600 nm_ of 0.5. After overnight incubation at 37°C and 5% CO_2_, inhibition zones were measured.

### Antiviral activity

#### Human immunodeficiency virus-1

Virus stocks of CCR5-tropic HIV-1 NL4-3 were generated by transient transfection of HEK293T cells with proviral DNA as described (Münch et al., 2007). Transfection mixture was replaced by 2 ml DMEM supplemented with 2 mM L-glutamine, 100 units/ml penicillin, and 100 μg/ml streptomycin and 2.5% heat-inactivated FCS after 8 h of incubation. 48 h later virus was collected by centrifuging the cell supernatant to remove cell debris for 3 min at 300 g. Virus stocks were stored at –80°C. For the infection assay, 10.000 TZM-bl cells were seeded the day before into 96-well-flat-bottom plates. Before infection, 80 μl of cell medium was added to the wells. Virus treatment experiments were done by mixing 70 μl of the peptide sample with 70 μl of 1/20 diluted HIV-1 for 1 h at 37°C. Then, 40 μl of the peptide-virus mix were added to each well. Two days post infection, the rates of infection were measured by Gal−Screen β−Galactosidase Reporter Gene Assay System for Mammalian Cells (Thermo Fisher Scientific) and the Orion II microplate luminometer (Berthold Technologies GmbH & Co., KG, Bad Wildbad, Germany). Values were corrected for the background signal derived from uninfected cells and antiviral effect of the peptide was then calculated by normalization to untreated cells which were set as 100% infection.

#### Herpes Simplex virus-1 and Herpes Simplex virus-2

Recombinant eGFP-encoding Herpes-Simplex-Virus 2 (Strain 333) was kindly provided by Patricia Spear (Northwestern University, United States) (Taylor et al., 2007) and HSV-1-GFP (Strain F) was provided by Benedikt Kaufer (Free University of Berlin). Virus stocks were generated by infecting 70–80% confluent Vero E6 cells in 175 cm3 cell-culture flasks in 30 ml cell medium (DMEM (supplemented with 2.5% heat-inactivated FCS, 2 mM L-glutamine, 100 units/ml penicillin, 100 μg/ml streptomycin, 1 mM sodium pyruvate, and 1x non-essential amino acids). Virus was harvested after 2–4 days as described above. For the infection assay, 5.000 ELVIS cells were seeded the day before into 96-well-flat-bottom plates. Before infection, the cell medium was removed and 80 μl of X-vivo cell medium supplemented with 2 mM L-glutamine, 100 units/ml penicillin, and 100 μg/ml streptomycin was added. Virus treatment experiments were done by mixing 35 μl of the peptide sample with 35 μl of HSV-1 or HSV-2 for 1 h at 37°C. Then, 20 μl of the peptide-virus mix were added to each well. For the experiments, HSV-1-GFP, HSV-2-GFP were used at a MOI of 0.05. Two days post infection, the rates of infection were measured by Gal−Screen β−Galactosidase Reporter Gene Assay System for Mammalian Cells (Thermo Fisher Scientific) and the Orion II microplate luminometer (Berthold Technologies). Values were corrected for the background signal derived from uninfected cells and antiviral effect of the peptide was then calculated by normalization to untreated cells which were set as 100% infection.

### Antifungal activity

A resazurin assay was used to detect the antifungal effect of Angicin on the viability of *Candida* cells. Therefore, 2.5 × 10^3^ cells were incubated in 200 μl RPMI-1640 medium with 300 mg/l L-glutamine and two different Angicin concentrations (25 μg/ml, 100 μg/ml) at 37°C for 24 h in a flat-bottomed polystyrene microtiter plate with 96 wells (Sarstedt AG & Co., KG, Nümbrecht, Germany) with shaking at 900 rpm on an Eppendorf shaker. For the following quantification of viable cells, a Resazurin-Reduction-Assay was performed. In brief, 20 μl of 0.15 mg/ml resazurin (Sigma-Aldrich) solution was added per well and incubated for 2 h at 37°C while shaking at 900 rpm. Fluorescence measurement (excitation wavelength 535 nm, emission wavelength 595 nm) of the resulting resorufin (viable cells are able to reduce resazurin to resorufin) was then performed by using a Tecan infinite F200 microplate reader (Tecan Group). The resulting data were normalized to the untreated control and the efficacy of Angicin was determined.

### Cytotoxicity

THP-1 cells were seeded in a 96-well plate (Thermo Scientific- Nunclon ™ Delta Surface) with a final cell number of 10^5^ cells per well. Angicin was diluted in cell culture medium (see Human cell lines and culture conditions) and added to cells in concentrations ranging from 100 to 12.5 μg/ml. THP-1 cells were incubated for 24 h and subsequently centrifuged for 10 min, 1,000 × g. 25 μl of the supernatant were discarded and replaced by 10% of Alamar blue™ HS cell viability reagent (Invitrogen AG, Waltham, MA) diluted in cell culture medium. After 1 h of incubation at 37°C and in the dark, absorbance at 572 and 600 nm was determined in a Tecan infinite M200 microplate reader (Tecan Group). The ratio of 572 nm to 600 nm was calculated. As positive control, cells treated with 1% Trition-X-100 (Sigma-Aldrich) were used, whereas untreated cells were the negative control.

Vero E6, ELVIS and TZM-bl cells were seeded in 96-well plates in 100 μl medium. The next day, peptide was added at indicated concentration and cell viability was quantified after 48 h with the MTT (3-(4,5-dimethylthiazole-2-yl)-2,5-diphenyl tetrazolium bromide)-based assay. Briefly, the medium was removed and 90 μl PBS and 1 μl MTT (5 mg/ml in PBS, Sigma-Aldrich) solution were added per well. Following a 2.5 h incubation time at 37°C, supernatant was discarded, and the formazan crystals were dissolved in 100 μl 1:1 DMSO-EtOH solution. Absorption was measured at 450 nm and baseline was corrected at 650 nm using a Vmax kinetic microplate reader (Molecular Devices, San Jose, CA). Untreated controls were set to 100% viability.

### Bioinformatic and statistical analysis

The GenBank database^1^ was accessed to obtain the nucleotide sequence of the Man-PTS operon of *L. monocytogenes* (accession number AF397145). Viewing and translation of nucleotide sequences was conducted using SnapGene 5.0 (from Inightful Science, San Diego, CA, United States; available at snapgene.com). For statistical analysis as well as to create graphs GraphPad Prism V6 (GraphPad Software, La Jolla, CA, United States) was used. To determine significant differences between treated and untreated cells Mann-Whitney *U*-tests were performed, using the GraphPad Prism V6 software.

## Results

### Kinetics of antimicrobial activity

**FIGURE 1:**
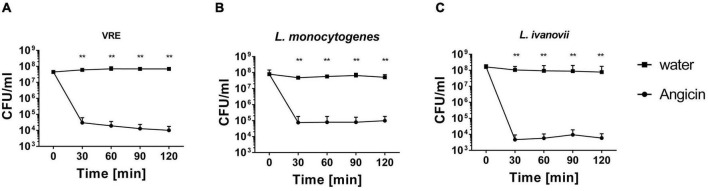
Survival of vancomycin resistant *Enterococcus faecium* (VRE) **(A)**, *Listeria monocytogenes* **(B)** and *Listeria ivanovii* **(C)** in the presence of Angicin. Bacteria were incubated with or without 1.56 μg/ml Angicin in 10 mM Phosphate buffer for 2 h. After 30, 60, 90, and 120 min, samples were taken and colony forming units (CFU) per ml were determined. To determined the amount of bacteria in the solution, a sample of the water control was used. Depicted is the mean + standard deviation of 5 independent experiments. Significant differences were calculated using a Mann-Whitney-*U*-test. **Illustrates a *p*-value < 0.01.

Angicin has been shown to inhibit growth of a variety of different bacteria, including streptococci, listeria and enterococci in RDAs (Vogel et al., 2021). To determine if Angicin can exert rapid bacterial killing in liquid culture medium, a survival assay against VRE, *L. monocytogenes* and *L. ivanovii* was performed (Figure 1). In this assay, bacterial cells at a density of 10^7^–10^8^CFU/ml were exposed to 1.56 μg/ml Angicin in phosphate buffer at 37°C for 30, 60, 90, and 120 min and subsequently cultured on THY plates. For all three species, a significantly reduced viability could be demonstrated. After 30 min of incubation with Angicin less than 1% of bacterial cells survived in this experimental setting. The greatest decline in bacterial cell numbers was seen at the earliest time point (30 min), indicating a fast mechanism of action.

### Mode of action

**FIGURE 2:**
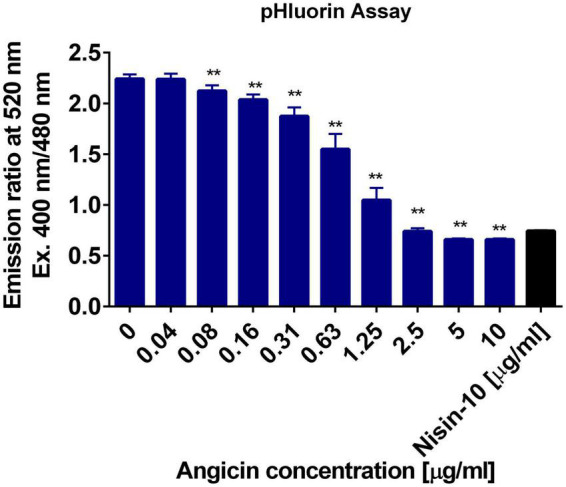
Membrane damaging activity of Angicin on *Listeria monocytogenes*. *L. monocytogenes* EGD-e/pNZ-P_help_-pHluorin was incubated with Angicin concentrations ranging from 0.04 to 10 μg/ml in LMB, pH 6.2. Untreated bacteria were used as a negative control and Nisin (10 μg/ml) treated bacteria as positive control. Depicted is the mean + standard deviation of 2 independent experiments, each with three independent cultures. A Mann-Whitney-*U*-test was used to calculate significant differences to the negative control, with ** showing a *p*-value < 0.01.

The mechanism of action of many bacteriocins is pore formation (Moll et al., 1999; Kumariya et al., 2019). We applied the improved pHluorin assay to investigate disruption of membrane integrity of *L. monocytogenes* (Reich et al., 2022). The assay is based on the constitutive expression of the biosensor pHluorin2, a GFP derivate with two distinct excitation peaks that change in fluorescence intensity dependent on the pH (Mahon, 2011). Thereby, the intracellular pH can be monitored and changes in intracellular pH indicate the disruption of membrane integrity of *L. monocytogenes.* Angicin concentrations ranging from 0.04 to 10 μg/ml were administered in this assay and the pore forming bacteriocin (Wiedemann et al., 2004), Nisin, was used as a positive control. 10 μg/ml Angicin showed the same effect on listerial membrane integrity as 10 μg/ml Nisin, implying that Angicin may have a similar mode of action as Nisin (Figure 2). Even a concentration of 0.08 μg/ml Angicin (approx. 119 nM) still interfered with membrane integrity in this assay, further highlighting its activity in the nanomolar range.

**FIGURE 3:**
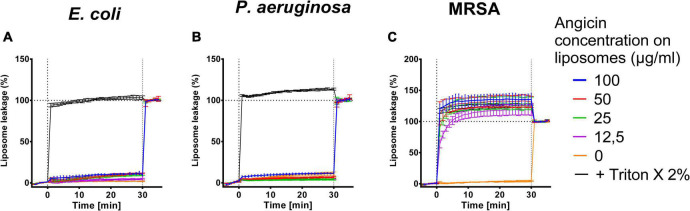
Effect of Angicin on bacteria derived liposomes. Liposomes filled with 50 mM carboxyfluorescein formed from bacterial lipid extracts either commercially purchased (*E. coli*, Avanti Lipids, **A**), or extracted from live bacteria [*P. aeruginosa* **(B)**, methicillin resistant *Staphylococcus aureus*, MRSA **(C)**] by the Folch method were added to 96-well plates and baseline fluorescence was recorded once per minute for 5 min in total once per minute. Angicin was then added at indicated concentrations and fluorescence increase (indicating membrane disruption) was recorded for 30 min with one measurement per minute. Triton X-100 was then added to all wells at 2% final concentration to induce total lysis. Background signal was subtracted (signal of liposome prior to addition of compounds) and normalized to fluorescent signal achieved in each well after complete liposome leakage induced by addition of 2% (v/v) Triton X-100. Depicted is the mean of three experiments ± standard deviation.

For further characterization of the antibacterial effect of Angicin, liposomes containing bacterial lipids were generated. These comprised lipid extracts of MRSA, *E. coli*, and *P. aeruginosa.* The liposomes contain carboxyfluorescin, which is only released upon liposome destruction, therefore an increase in fluorescence signal indicates membrane disruption. *E. coli* derived liposomes were nearly unaffected by an Angicin treatment (Figure 3). The highest Angicin concentration applied (100 μg/ml) caused only an 9.8% leakage after 30 min, compared to a leakage of 1.8% for untreated liposomes. For *P. aeruginosa* derived liposomes, no effect was seen, with 100 μg/ml Angicin leading to a leakage of 11.53% and untreated liposomes showing a leakage of 10.8%. Liposomes derived from MRSA showed the highest sensitivity toward Angicin. All tested concentrations were able to destroy liposomes completely. Taken together, these data confirm the previously observed activity of Angicin against Gram positive bacteria, while Gram negative organisms are resistant.

### Receptor specificity

**FIGURE 4:**
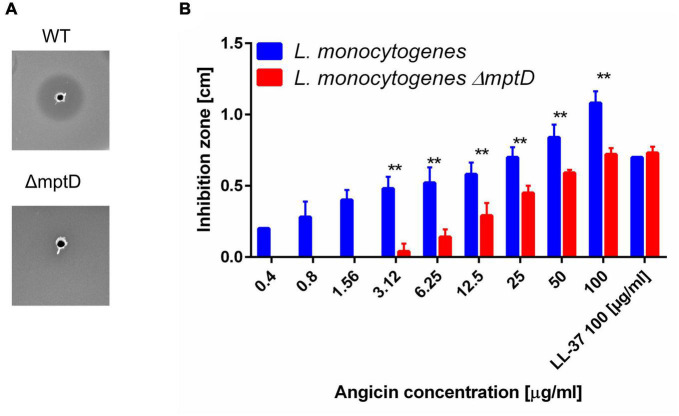
Receptor specificity of Angicin activity. *L. monocytogenes* EGDe and its isogenic mutant EGDeΔ*mptD* were used as target strains in a radial diffusion assay. Inhibition zones were quantified in cm, the AMP LL-37 served as positive control. Depicted is the mean + standard deviation of 5 independent experiments. **(A)** Mann-Whitney-*U*-test was performed to determine significant differences between the inhibition zone size of the wildtype or the mutant strain. A *p*-value < 0.01 is illustrated by **. **(B)** Picture of a one-layer RDA conducted with *L. monocytogenes* EGDe or its isogenic mutant EGDeΔ*mptD* as target strains and *S. anginosus* BSU 1211 as bacteriocin producer.

Many bacteriocins bind to a receptor for efficient inhibition of target bacteria. In listeria and other Gram positive organisms the Man-PTS, especially the subunits IIC and IID have been identified previously as receptors for class IId bacteriocins (Tymoszewska et al., 2018; Jeckelmann and Erni, 2020). If Angicin interacts with this system is, however, unknown. To investigate whether Angicin targets the Man-PTS, the susceptibility of a *L. monocytogenes* mutant containing an 84 bp deletion (amino acids 219–246) in subunit IID (*L. monocytogenes* Δ*mptD*) was investigated (Dalet et al., 2001). In a RDA the Angicin-producing strain *S. anginosus* BSU 1211 was not able to cause an inhibition of *L. monocytogenes*Δ*mptD* while the respective listerial wildtype strain showed an inhibition zone of 0.67 ± 0.07 cm (Figure 4A). Furthermore, the activity of synthetic Angicin against *L. monocytogenes*Δ*mptD* was assessed (Figure 4B). In accordance with the previously obtained results, the *L. monocytogenes*Δ*mptD* mutant showed a significantly decreased sensitivity toward synthetic Angicin, when compared to the wildtype. To verify that this effect was specific for Angicin, the susceptibility of *L. monocytogenes*Δ*mptD* toward other antilisterial peptides, like β2-microglobulin (B2M) or Cm-p5 was examined (Holch et al., 2020; González-García et al., 2021, p. 5; Supplementary Figure 1). However, inhibition zones against *L. monocytogenes* and its isogenic *mptD* mutant were identical for these AMPs. In summary, our data supports the conclusion that, although the deletion mutant is still affected, the main receptor for Angicin is the Man-PTS of listeria.

### Antimicrobial activity of Angicin

**FIGURE 5:**
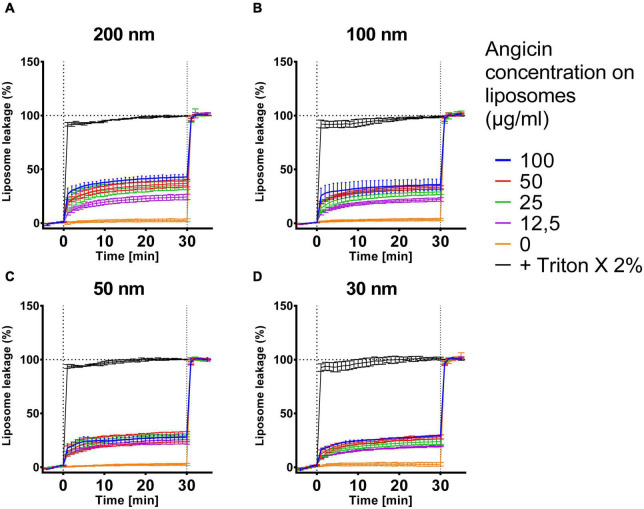
Effect of Angicin on virus-like liposomes. Liposomes that resemble virus particles with sizes of 200 nm **(A)**, 100 nm **(B)**, 50 nm **(C)** or 30 nm **(D)** containing 50 mM carboxyfluorescin were added to 96-well plates and baseline fluorescence was recorded once per minute for 5 min. Angicin was then added at indicated concentrations and fluorescence increase (indicating membrane disruption) was recorded for 30 min with one measurement per minute. Triton X-100 was then added to all wells at 2% final concentration to induce total lysis. Background signal was subtracted (signal of liposome prior to addition of compounds) and normalized to fluorescent signal achieved in each well after complete liposome leakage induced by addition of 2% (v/v) Triton X-100. Depicted is the mean of two to three experiments ± standard deviation.

Bacteriocins are sometimes not only able to inhibit bacteria but furthermore viruses and fungi can be suppressed (Al Kassaa et al., 2014; Dehghanifar et al., 2019). To further characterize the spectrum of antimicrobial activity of Angicin, we analyzed its effect on biomimetic liposomes of different sizes containing lipids chracteristic of viral particles with a liposome leakage assay (Figure 5). In this setting, the effect of Angicin on 200 nm liposomes was the most pronounced, with a maximum leakage of 42.9% when treated with 100 μg/ml Angicin. A treatment with 12.5 μg/ml Angicin still caused a leakage of 24.3%. Percentage of leakage for 100 nm liposomes ranged between 38.15 and 22.03%, for 50 nm liposomes between 28.15 and 23.45% and for 30 nm liposomes between 29.25 and 19.65%. In all cases, Angicin never caused a complete destruction of liposomes, rather a plateau was reached after an initial rapid increase in liposome leakage in the first 1–2 min after treatment start.

Next, the antiviral activity of Angicin against human immunodeficiency viruses-1 (HIV-1), herpes simplex virus-1 (HSV-1) and herpes simplex virus-2 (HSV-2) was investigated (Supplementary Figure 2). After preincubation of virus with Angicin, ELVIS or TZM-bl cells were infected with HSV-1 and HSV-2 or HIV-1, respectively. Two days post infection, infection rates were determined, demonstrating that Angicin had no antiviral activity (Supplementary Figure 2). Furthermore, antifungal activity was investigated. Angicin did not influence the viability of planktonic *Candida albicans, Candida auris*, and *Candida parapsilosis* cells (Supplementary Figure 3). To sum up, Angicin is a potent antibacterial peptide, but it has neither an antiviral nor an antifungal activity.

### Angicin cytotoxicity

**FIGURE 6:**
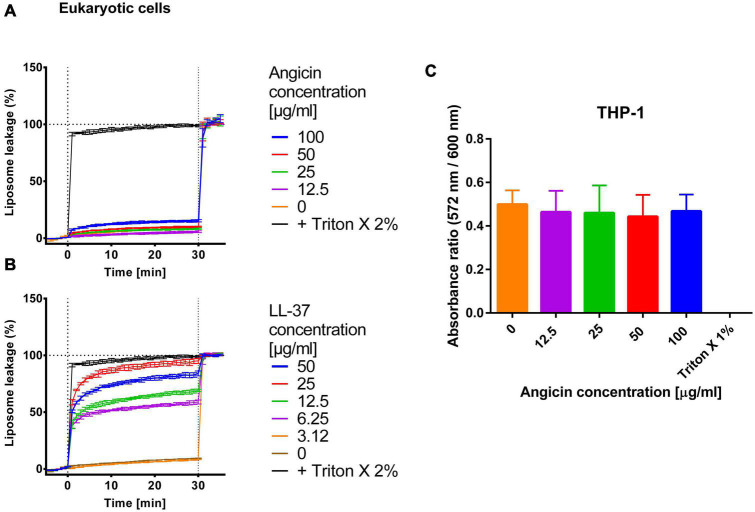
Effect of Angicin on liposomes and cellular metabolic activity. For liposomes derived from eukaryotic cells baseline fluorescence was measured once per minute for 5 min. Subsequently, Angicin **(A)** or the AMP LL-37 **(B)** was added at indicated concentrations and fluorescence increase (indicating membrane rupture) was recorded every minute for 30 min. Triton X-100 was added at a final concentration of 2% to induce total lysis. Background signal was subtracted (signal of liposome prior to addition of compounds) and normalized to fluorescent signal achieved in each well after complete liposome leakage induced by addition of 2% (v/v) Triton X-100. Depicted is the mean ± standard deviation. Three independent experiments were performed. **(C)** Monocytic THP-1 cell were incubated for 24 h with the indicated Angicin concentrations. Afterward the metabolic activity of cells was measured with a Resazurin assay. A treatment with Triton X was used as a positive control and untreated cells as negative control. A Mann-Whitney-*U*-test was conducted to test for significant differences. Depicted are mean and standard deviation of five independent experiments each performed in triplicate.

In a SYTOX Green permeabilization assay it has previously been shown that Angicin destroys bacterial cell membranes (Vogel et al., 2021). However, the interaction of Angicin with isolated lipid bilayers has not been studied. To investigate whether Angicin damages the cellular membranes of eukaryotic cells, a liposome leakage assay was performed. Liposomes derived from eukaryotic cells were incubated with different Angicin concentrations, ranging from 12.5 to 100 μg/ml. Angicin did not cause membrane leakage at concentrations of up to 50 μg/ml and only resulted in a modest leakage as compared to background at 100 μg/ml (Figure 6A). As a control, eukaryotic cells were incubated with the cytotoxic AMP LL-37 (Gudmundsson et al., 1996; Svensson et al., 2017). Here, a clear cytotoxic effect on cells can be observed (Figure 6B). Additionally, cytotoxicity assays were performed on monocytic THP-1 cells, Vero E6, ELVIS and TZM-bl cells. Measurement of metabolic activity by a Resazurin assay (THP-1) showed no significantly reduced cell viability when compared to untreated cells (Figure 6C). Similarly, Angicin at concentration of up to 40 μg/ml had no significant effect on metabolic activity in Vero E6, ELVIS and TZM-bl cells, which were analyzed by MTT assay (Supplementary Figure 4). Taken together, this data demonstrates that Angicin is not cytotoxic.

## Discussion

While *S. anginosus* is commonly found as a commensal of mucosal membranes, it can also cause severe invasive infections such as brain abscesses, bacteremia and respiratory infections. Angicin is the first identified and characterized bacteriocin of *S. anginosus* (Vogel et al., 2021). It is active against multiple Gram positive bacteria including other *S. anginosus* strains and various streptococcal species as well as listeria and VRE (Vogel et al., 2021). Due to this wide spectrum of activity, Angicin is an interesting candidate for clinical applications. This study demonstrates that synthetic Angicin is a potent antibacterial agent. In liquid cultures, 1.56 μg/ml Angicin reduce viable cells to under 1% within 30 min (Figure 1), illustrating not only a high potency but also a fast mechanism of action. The fast mechanism of action of Angicin is further confirmed by the liposome assay, which shows an immediate leakage of fluorescent dye following Angicin exposure (Figure 3). This could be observed for liposomes resembling virus like membranes and MRSA lipid extracts. Interestingly, in previous experiments MRSA was not sensitive toward Angicin (Vogel et al., 2021), while MRSA derived liposomes are highly susceptible to membrane damage caused by Angicin. A possible explanation is, that during the experimental procedure only lipids, but not the peptidoglycan layer is extracted, which may be rendering the produced liposomes more sensitive toward membrane disrupting agents. It is possible, that in the absence of peptidoglycan layers, the electrostatic interaction between cationic Angicin and the negatively charged membrane is sufficient for Angicin to insert into the membrane and form a pore. Gram negative species are less effected, since they only harbor a thin peptidoglycan layer. It needs to be highlighted that MRSA derived liposomes are not a perfect representation of a MRSA bacterial cell, which might further explain the differences in susceptibility.

AMPs can target bacteria through various mechanisms, including disruption of membrane integrity, interaction with intracellular targets or indirectly by modulating host defense systems (Mahlapuu et al., 2016). The most probable mechanism of action for Angicin is pore formation, which is characterized by leakage of ions and metabolites. This causes a disruption of membrane potential, intracellular pH homeostasis and proton motive force, ultimately leading to cell death (Christensen and Hutkins, 1992; Minahk et al., 2000). Pore formation caused by Angicin was previously indicated by a SYTOX Green membrane permeabilization assay (Vogel et al., 2021) and was here further substantiated by liposome leakage assays as well as a pHluorin assay. Here, the pH-sensitive GFP-derivate pHluorin2 is used to monitor intracellular pH of *L. monocytogenes*, which allows indirect determination of membrane integrity (Reich et al., 2022). In the pHluorin assay, Angicin was as active as Nisin, which is a bacteriocin known for its pore forming capabilities (Wiedemann et al., 2004). The pHluorin-assay showed that concentrations of 2.5 μg/ml were sufficient to completely perforate *L. monocytogenes* cells (Figure 2). In contrast, for the SYTOX Green membrane permeabilization assay the tenfold Angicin concentration was required to measure membrane disruption (Vogel et al., 2021). The pHluorin-assay is a sensitive tool to measure membrane disruption and the results demonstrate that Angicin is a potent antibacterial agent with activity in the nanomolar range.

Many bacteriocins utilize a receptor for efficient killing of target cells. The Man-PTS has been identified previously as a receptor for streptococcal bacteriocins and may thus also represent the target of Angicin (Hossain and Biswas, 2012; Oftedal et al., 2021; Vogel et al., 2021). A first hint in this direction is that organisms like staphylococci, *Bacillus subtilis* and *Candida albicans* that are insensitive toward Angicin do not harbor a Man-PTS system (Tymoszewska et al., 2017). In viruses and in eukaryotic membranes the Man-PTS is absent as well, explaining their insensitivity toward Angicin. Furthermore, a deletion in *mptD* gene rendered *L. monocytogenes* cells insensitive toward *S. anginosus* BSU 1211 in our experiments (Figure 4). This deletion of 28 amino acids (219–246) occurred in the gamma loop of subunit IID (Dalet et al., 2001). In line with this, the *mptD* mutant also showed increased resistance toward synthetic Angicin, supporting the interpretation that the Man-PTS is the receptor for Angicin. The *mptD* gene encodes for the subunit IID of the Man-PTS, which all in all consists of four subunits. Subunits IIA and IIB are intracellular and IIC and IID are localized in the membrane. The Man-PTS is recognized as the receptor for especially class II bacteriocins including for example pediocin PA-1 isolated from *Pediococcus acidilactici* (class IIa), mesentericin Y105 from *Leuconostoc mesenteroides* (class IIa), Garvicin Q from *Lactobacillus garvieae* (class IId) and class IIe bacteriocin microcin MccE492 from *Klebsiella pneumoniae* (Dalet et al., 2001; Ramnath et al., 2004; Biéler et al., 2010; Tymoszewska et al., 2018; Jeckelmann and Erni, 2020). For inhibition by bacteriocins subunits IIC and IID are important (Dalet et al., 2001; Ramnath et al., 2004; Tymoszewska et al., 2018, 2020). For example, class IIa bacteriocins inhibit various species, including listeria, lactobacilli and enterococci but not lactococci (Kjos et al., 2009). The activity of these peptides is dependent on a single extracellular loop of subunit IIC (Kjos et al., 2010). In contrast, class IId bacteriocins like Lactococcin A inhibit *Lactococcus lactis* strains, and for its species-specific activity both the subunit IIC and IID are needed (Holo et al., 1991; Diep et al., 2007). The activity of the class IId bacteriocins BacSJ and Garvicin Q also depends on subunits IIC and IID. Garvicin Q resistant mutants either expressed a prematurely truncated subunit IIC or IID or contained missense mutations in one of these subunits (subunit IIC: Pro100 → His, subunit IID: Thr123 → Ile and Pro111 → Ser) (Tymoszewska et al., 2017). Furthermore, six different missense mutations in either the subunit IIC (Gly62→Val) or IID (Pro123 →His, Arg200→His, Leu83→Phe, Phe226→Ser, Leu197→Phe) led to an at least eightfold decreased sensitivity of *L. lactis* toward BacSJ. Many of these *L. lactis* mutants also showed increased resistance toward Garvicin Q (Tymoszewska et al., 2020). However, the mutant carrying the Leu83→Phe mutation showed no altered sensitivity toward Garvicin Q. This indicates that different bacteriocins utilize different amino acids for an efficient interaction with the Man-PTS. Additionally, bacteriocins targeting the Man-PTS show different spectra of activity and have distinct amino acid sequences, giving further support to the theory of a differential interaction of the different bacteriocins with the Man-PTS (Holo et al., 1991; Ramnath et al., 2004; Tymoszewska et al., 2018, 2020). Thus, explaining why *L. monocytogenes*Δ*mptD* is still affected by Angicin. By deleting these 28 amino acids of subunit IID an interaction between the Man-PTS and Angicin is disturbed, but Angicin might still be able to interact with amino acids outside of the deleted region. As another explanation Angicin may bind to both subunits, IID and IIC. Subunit IIC is not affected by the deletion in *L. monocytogenes*Δ*mptD* and an interaction with Angicin may still be possible. This interaction would then be less efficient, leading to reduced inhibition zones. Even though the role of Man-PTS as a receptor for various bacteriocins is well established, the exact mechanism of action remains to be elucidated. Currently, two models for pore formation exist (Ríos Colombo et al., 2018). Either the pore is formed by the bacteriocins themselves after an initial docking to the Man-PTS as it is proposed for Enterocin CRL35 or the bacteriocin binds to the Man-PTS and thereby induces structural changes of subunit IIC leading to an opening of the intrinsic pore as it is proposed for Garvicin Q (Barraza et al., 2017; Tymoszewska et al., 2018; Farizano et al., 2019).

Membrane disrupting bacteriocins may display cytotoxicity against human cell lines, limiting future applications as antimicrobial agents. Therefore, Angicin cytotoxicity toward eukaryotic cells was determined, but could not be detected (Figure 6 and Supplementary Figure 4). The virus membrane is closely related to eukaryotic membranes (Lenard and Compans, 1974); thus, it makes sense that in the liposome leakage assay also virus like particles show a high resistance toward Angicin induced leakage. In eukaryotic cells as well as in virus like particles the here identified receptor, the Man-PTS, is not present, explaining the low susceptibility of these cells toward Angicin.

To sum up, Angicin is a potent antibacterial peptide that forms pores in its target cells and uses the Man-PTS system as a receptor, which is a novel finding. It displays a strong antimicrobial activity against various Gram-positive bacterial pathogens, based on a rapid disruption of bacterial membranes, while eukaryotic membranes remain undamaged.

## Data availability statement

The raw data supporting the conclusions of this article will be made available by the authors, without undue reservation.

## Author contributions

VV and BS designed the study and analyzed the data. MJ performed survival assay experiments under the supervision of VV. L-RO conducted liposome leakage assays, viral infection assay, and cytotoxicity assays under the supervision of JM. MH analyzed the antifungal activity of Angicin under the supervision of A-KK and FR. VV conducted radial diffusion assays and cytotoxicity assays of THP-1 cells and wrote the manuscript. VV performed the pHluorin-assay under the supervision of SR and CR. BS reviewed the manuscript. All authors contributed to the article and approved the submitted version.

## Footnotes

1
http://www.ncbi.nlm.nih.gov/
