# Supplementary material for: The bacteriocin Angicin interferes with bacterial membrane integrity through interaction with the mannose phosphotransferase system
Source: Front Microbiol. 2022 Sep 6;13:991145. doi: 10.3389/fmicb.2022.991145 (PMC9486217; doi:10.3389/fmicb.2022.991145)
Supplement: Supplementary file 1 [file Table_1.DOCX]

Supplementary Material

Content

[**Figure S1: Effect of a deletion in the *mptD* gene of *L. monocytogenes* on susceptibility towards antimicrobial peptides. 2**](#_Toc109730309)

[**Figure S2: Effect of Angicin on viral infection. 3**](#_Toc109730310)

[**Figure S3: Effect of Angicin on the viability of different *Candida spp.* 4**](#_Toc109730311)

[**Figure S4: Cytotoxicity of Angicin on eukaryotic cells. 5**](#_Toc109730312)





# Figure S1: Effect of a deletion in the *mptD* gene of *L. monocytogenes* on susceptibility towards antimicrobial peptides. *L. monocytogenes* EGDe or its isogenic mutant EGDeΔ*mptD* were used as target strains in a radial diffusion assay: They were tested for their susceptibility towards β2-Microglobulin (B2M) (A) or Cm-p5 (B). Visualized is the mean + standard deviation of 5 independent experiments. By a Mann-Whitney-U test, no significant differences could be determined.





# Figure S2: Effect of Angicin on viral infection. Human immunodeficiency viruses-1 (HIV-1) (A), herpes simplex virus-1 (HSV-1) (B) or herpes simplex virus-2 (HSV-2) (C) were diluted and preincubated for 1h incubation at 37 °C with different Angicin concentrations (ranging from 0.0064 to 100 µg/ml). Afterwards, TZM-bl or ELVIS cells respectively were infected with the Angicin-virus mixture. Infection rates were determined two days post infection by measuring β-galactosidase activity of the reporter cell lines. Values were baseline-subtracted and normalized to cells infected without the addition of compound. Shown is the mean + SD of one experiment performed in triplicates





# Figure S3: Effect of Angicin on the viability of different *Candida spp.* *Candida albicans, Candida parasilosis* and *Candida auris* were incubated with 25 or 100 µg/ml Angicin and after 24 h of incubation, viability was checked in a resazurin assay. Values were normalized to untreated cells. Depicted is the mean of three technical replicates.





# Figure S4: Cytotoxicity of Angicin on eukaryotic cells. Angicin was serially diluted in PBS, before the addition to the indicated cell lines. Two days post addition, the metabolic activity of the cells was determined using the MTT assay. Values were baseline-subtracted and normalized to untreated cells. Shown is the mean + SD of one experiment done in triplicates.
